# Transient Expression of Hen Egg White Lysozyme (EWL) in *Nicotiana benthamiana* Influences Plant Pathogen Infection

**DOI:** 10.3390/life15040642

**Published:** 2025-04-14

**Authors:** Zhuo Meng, Haijuan Wang, Chongyi Jia, Guihua Chen, Mingmin Zhao

**Affiliations:** 1 College of Horticulture and Plant Protection, Inner Mongolia Agricultural University, Hohhot 010018, China; mengzhuo688@163.com (Z.M.); wanghaijuan9264@163.com (H.W.); 2 Bayannur Agriculture and Animal Husbandry Bureau (Rural Revitalization Bureau), Bayannur City 015000, Mongolia; 13847832336@163.com

**Keywords:** hen egg white lysozyme, *Nicotiana benthamiana*, turnip mosaic virus, tobacco mosaic virus, *Botrytis cinerea*

## Abstract

Lysozyme is an enzyme that hydrolyzes bacterial cell walls, which is functional for destroying the integrity of bacteria, enhancing the activity of immune cells, participating in immune signal transmission, helping to maintain the micro-ecological balance of the gastrointestinal tract, etc. Egg white lysozyme (EWL), as one of the typical representatives of lysozyme, is the most widely used enzyme in production so far, and is also one of the most complex structures of lysozyme. EWL also helps protect plants from fungal and bacterial diseases. Here, we report the effect of EWL on infections from plant viruses. The EWL gene was cloned and characterized. The EWL protein sequence analysis identified a conserved domain of lysozyme activity and the sharing of a 100% identical EWL protein from the *Coturnix japonica* lysozyme. Then, the EWL gene was cloned into the plant expression vector pEAQ-HT-DEST3 and transiently expressed in *Nicotiana benthamiana (N. benthamiana)*. We found that EWL expression in *N. benthamiana* significantly contributed to infections by the turnip mosaic virus (TuMV) but not by the tobacco mosaic virus (TMV). Plants that transiently expressed EWL showed an obvious increase in resistance to *Botrytis cinerea (B.cinerea)*. Our results suggested a new research point for the application of EWL on plant pathogen infections.

## 1. Introduction

Lysozyme, also known as muramidase or N-acetylmuramide glycanohydrlase [1], is an alkaline enzyme that hydrolyzes mucopolysaccharides in bacteria [2]. Lysozyme is divided into plant lysozyme, egg white lysozyme, animal lysozyme and so on [3]. Lysozyme in the egg white content is very high, about 3–4% of the total protein content [4]. According to the differences in amino acid sequences and substrates of lysozyme [5], lysozyme can be broadly divided into three types: C-type (hen egg white lysozyme), G-type (goose egg white lysozyme), and phage lysozyme. To date, the most widely used enzyme in production is hen egg white lysozyme, which, as a typical representative of lysozyme, is one of the most structurally understood lysozymes [6,7].

Lysozyme is an important component of the innate immune system of animals and is found mainly in mammalian milk, saliva, tears, and mucus [8,9], as well as in white blood cells [10,11]. In livestock production, it has various functions such as antibacterial, anti-inflammatory, tissue repair, and improving animal immunity. It can treat mastitis in dairy cows [12,13]. Lysozyme plays an important role in human health and can inhibit colitis by regulating human intestinal flora. It has been widely used in food preservation, biomedicine, and baby food additives [14,15]. Lysozyme is one of the most important components of the skin’s protective barrier. Insufficient secretion of lysozyme can cause various skin diseases [15]. Therefore, there are many studies related to the use of lysozyme in the treatment of traumatic infections and skin diseases. With the increasing research on the mechanism of bacterial inhibition and the optimization of lysozyme’s activity, the application of hen egg white lysozyme (EWL) in food preservation, freshness preservation, and enhancement of the nutritional value of food is becoming more and more promising.

Particularly, lysozyme has a variety of benefits for both livestock production and human health, and it can also make plants more resistant to pathogenic bacteria. A lysozyme derived from *Phaseolus vulgaris* is highly resistant to *Alternaria alternata*, *Pythium aphanidermatum*, *Fusarium oxysporum*, and *Botrytis cinerea* [16]. A quantity of 10 ug lysozyme from cauliflower has an inhibitory effect on bacteria like *Xanthomonas campestris* and *Erwinia carotovora*, exhibiting 5.90 and 3.88-fold inhibition, respectively. Lysozyme also has strong resistance to *Fusarium solani*, *Leptosphaeria maculans*, *Botrytis cinera*, *Curvularia lunata*, *Rhizoctonia solani*, and *Alternaria alternata* [17]. By rupturing the β-1,4 glycosidic bond in the structure of the cell walls among N-acetylcytidylic acid and N-acetylglucosamine, lysozyme primarily lyses bacteria by converting the insoluble mucopolysaccharides into soluble glycopeptides, which causes the cell wall to rupture and its contents to leak out [2]. To render viruses inactive, lysozyme additionally forms compounds with DNA, RNA, and de-cohesion proteins and binds directly with negatively charged viral proteins. Currently, most of the research on the disease resistance of lysozyme mainly focuses on pathogenic fungi; however, lysozyme can effectively inhibit viral replication and play an antiviral role, as lysozyme has important antiviral properties. However, the protein structure analysis of EWL with other lysozymes and the effect on plant viral infections have not been reported yet [18].

Because *Nicotiana benthamiana* (*N. benthamiana*) is a typical plant model used for transient expression, it was also used to asses antiviral infection. In addition, it is very easy to grow and obtain large amounts of tissue from *N. benthamiana* plants, which will help expand the production of EWL, not by animals but rather by plants, for use in future applications. It is well known that lysozyme from hen egg whites displays obvious antifungal effects in plants. We would like to test if it can also play a role in antiviral resistance. This is why we have chosen hen egg white lysozyme. In the present study, an EWL gene was inserted into the plant expression vector pEAQ-HT-DEST3 by the Gateway recombinant system to create pEAQ-EWL. To evaluate the role of the EWL gene on plant viral infections, *Agrobacterium tumefaciens* (*A. tumefaciens*) harboring pEAQ-EWL was infiltrated into *N. benthamiana* leaves. Plant viruses, such as the turnip mosaic virus (TuMV) and the tobacco mosaic virus (TMV), or the fungal pathogen *Botrytis cinerea*, were used for inoculation into the infiltrated leaves. Our results suggested a new research point for the application of EWL on plant pathogen infection.

## 2. Materials and Methods

### 2.1. Experimental Materials

The vectors pDONR207 and pEAQ-HT-DEST3 from the Gateway system were supplied by Juan Antonio García’s lab (Centro Nacional de Biotecnologia, CSIC, Madrid, Spain). TuMV-GFP and TMV-GFP viral infection clones were described previously [19,20]. The hen egg white lysozyme gene was synthesized by Shanghai Plush Bio Co. (Shanghai, China).

### 2.2. N. benthamiana Plant Preparation

The *N. benthamiana* seeds were planted in soil and grown at 22 °C in a greenhouse with 16 h of light and 8 h of darkness. Agro-infiltration assays were carried out once the seedlings had produced 4–5 leaves, which had all grown in the same conditions [21].

### 2.3. Hen Egg White Lysozyme Gene Expression Vector Construction

Gene-specific primers were designed based on the EWL sequence (NM_205281.2) [22] (Table S1). A PCR reaction of 50 μL, including 2 μL primers, 5 μL 10× PCR buffer, 4 μL dNTP mixture, 1 μL EWL plasmids, and 0.25 μL Taq polymerase, was conducted. Pre-denaturation was performed at 95 °C for 5 s, followed by 29 PCR cycles (at 95 °C for 15 s, 67 °C for 30 s, 72 °C for 2 min, and 72 °C for 10 min). The products were run by a 1% agarose gel. An agar-gel DNA purification kit (Bevo Medical, Shexian, China) was used to recover and purify PCR fragments (EWL gene, 505 bp). The BP reaction was used to recombine the EWL gene and the pDONR207 vector at 25 °C for overnight. The reaction was transformed into *Escherichia coli* (*E. coli*) DH5α competent cells, which were then inoculated into an LB solid medium containing gentamicin and incubated at 37 °C for overnight. After being cultured for 8–12 h on an LB plate with gentamicin, positive clones were confirmed by PCR. Using *Pst*Ⅰ and *Eag*Ⅰ, the pDONR207-EWL plasmid was confirmed by restriction enzyme digestion with *Pst*Ⅰ and *Eag*Ⅰ. The binary expression vector for pEAQ-HT-DEST3-EWL was constructed by an LR reaction. The reaction contained 3.5 μL pDONR207-EWL (588.7 ng/μL), 1 μL LR Clonase™ II enzyme mixture, and 0.5 μL pEAQ-HT-DEST3 and was incubated at 25 °C for 8.5 h. Then, the reaction was transformed into *E. coli* DH5α competent cells, which were then cultured in an LB solid medium with kanamycin (50 mg/mL). The pEAQ-EWL-positive clones were verified by PCR and restriction enzyme digestion using *ApaL*Ⅰ and *Spe*Ⅰ.

### 2.4. In Silico Analysis of EWL Protein

ClustalW (https://www.genome.jp/tools-bin/clustalw) and SnapGene software (Version 6.0) were used for sequences alignment, and the evolutionary tree was profiled by MEGA11 [23]. The genetic relationship of EWL at the nucleotide level in different species was constructed. ExPaSy-ProtParam (https://web.expasy.org/protparam/) and NovoPro software (https://www.novopro.cn/tools/) were used for in silico analyses of the physicochemical properties, secondary structures, and conserved domains of the EWL proteins. Three-dimensional structural models of EWL were predicted using Swiss-Model [24].The sequence of conserved domains of the following proteins, including *Coturnix japonica* lysozyme (XP_015711651.1), *Danio rerio* lysozyme (AAK85299.1), *Bos taurus* lysozyme (ADZ96001.1), *Ovis aries* lysozyme (ABF20559.1), *Capra hircus* lysozyme (AHA61787.1), *Vicugna pacos* lysozyme (XP_031539227.1), *Homo sapiens* lysozyme (ACO37637.1), and *Bubalus bubalis* lysozyme (ABQ02304.1), were searched from the NCBI database. The structural similarity matrix between EWL and different species was obtained by TBtools software (Version 2.07) [25]. The structural tree diagram between the hen egg white lysozyme and lysozymes of different species was obtained using MEGA software (Version 11).

### 2.5. Transient Expression of EWL in N. benthamiana

The pEAQ-EWL construct was transformed into *A. tumefaciens* C58C1. This *A. tumefaciens* carrying the pEAQ-EWL plasmid was cultured overnight at 28 °C and then centrifuged at 4000 rpm for 15 min. The pellets were suspended up to OD_600_ = 1 with induction buffer containing 0.5 M MES buffer (pH 5.6), 1 M MgCl_2_, and 0.1 M acetosyringone. Then, agrobacteria were incubated at room temperature for 5 h. The leaves of 4–5-week-old *N. benthamiana* were infiltrated using a needleless syringe. An empty pEAQ-HT-DEST3 vector was used as the control. A quantity of 12 plants were used for each treatment, and there were three replicates for each experiment.

### 2.6. RT-qPCR

Total RNA was purified from samples by Trizol reagents and used as the cDNA RNA template. The reaction of RNA template denaturation and annealing was prepared using 1 μL OligodT (18T) Primer (50 μM), 1 μL dNTPMix (10 mM each), and 1 ug total RNA. A thermal cycling program was run (65 °C for 5 min) in order to denature and anneal the RNA template. The first strand of cDNA synthesis was performed with the reaction, including 10 μL of the reaction of RNA template denaturation and annealing, 4 μL dNTP Mix (10 mM each), 0.5 μL RNase Inhibitor (40 U/μL), 1 μL Evo M-MLV Plus RTase (200 U/μL), and up to 20 μL RNase-free water. Then, the reaction was incubated at 30 °C for 10 min, 42 °C for 30 min, and 95 °C for 5 min.

The gene-specific primers were designed as shown in Table S1. A PCR reaction was performed using MonAmp^™^ SYBR Green qPCR mix (Monad, Wuhan, China) in an FTC-3000P Real-Time Quantitative Thermal Cycler (Funglyn Biotech, Richmond Hill, ON, Canada). The PCR reaction contained 10 μL 2X SYBRGreen Pro TaqHS Premix (ROX plus), 0.4 μL Primer F (10 μM), 0.4 μL Primer R (10 μM), 100 ng Template, and up to 20 μL RNase-free water. The PCR cycle was run at 95 °C for 30 s for 40 cycles; and then at 95 °C for 15 s, 60 °C for 1 min, and 72 °C for 30 s. EWL expression was normalized using reference gene NbUBI. Fold changes relative to the control were calculated using the ΔΔ^CT^ method [21]. SPSS software (Version 23) was used for analyzing the significant differences.

### 2.7. The Influence of EWL Protein on Viral Infection

The constructed plasmid pEAQ-EWL was transformed into Agrobacterium C58C1. The Agrobacterium was collected in a 50 mL centrifuge tube at 4000 rpm for 15 min. The supernatant was discarded. A quantity of 20 mL of induction solution (0.01 M MgCl_2_, 0.01 M MES solution, 0.15 mM Aceto mixed solution) was added. The precipitate of the bacterium was suspended and washed in order to remove the residual antibiotics and other impurities in the bacterium and was then centrifuged at 4000 rpm for 10 min. The supernatant was discarded, and 2 mL of induction solution was added to the vortex and mixed. This solution was incubated for 5 h at room temperature. To reach OD_600_ = 1.0, the bacterial suspension was infiltrated into *N. benthamiana* leaves (4~5 leaves) with a 1 mL syringe. The pEAQ-EWL was co-injected with TMV-GFP and TuMV-GFP viral infection cloning with (3:1, *v*/*v*). pEAQ-HT-DEST3 was co-injected with TMV-GFP and TuMV-GFP viral infection cloning (3:1, *v*/*v*) as the control. This procedure was done to ensure that the leaves had been wholly infiltrated.

### 2.8. GFP Florescence Imaging

The viral symptoms were observed and recorded after 7 dpi. The GFP florescence imaging was done with a GFP imager (LUYOR−3104 Handheld NDT UV Lamp) with 360–370 nm. The systemic leaves of the plant were collected and stocked in −80 °C.

### 2.9. Western Blot

The plant samples were ground into powder in liquid nitrogen. A quantity of 0.1 g of this powder was added to 200 uL buffer protein extract buffer (pH = 7.5; 5% β-mercaptoethanol, 20% sodium dodecyl sulfate (SDS), 6 M urea, 125 mM Tris HCl, and a small amount of bromophenol blue). The mixture was incubated in a metal bath at 95 °C for 10 min and then on ice for 1 min. The tube was centrifuged at 12,000 rpm at 4 °C for 10 min. A quantity of 5 uL of obtained protein extracts were added into gel with sodium dodecyl sulfate–polyacrylamide and then subjected to electrophoresis.

The Geldoc image system (Thermo Fisher Scientific, Waltham, MA, USA) was used to take pictures of the protein gel and used as the loading control [26]. Then, the proteins were transferred to a nitrocellulose membrane and blocked for 2 h in a blocking solution. The nitrocellulose membrane was washed with TBST three times. Primary antibodies and anti-GFP antibodies for TuMV and TMV were used in immunodetection. Secondary antibodies were composed of mouse monoclonal antibodies and goat anti-rabbit IgG together with horseradish peroxidase (ab205718; Abcam, Cambridge, UK). After three washings with TBST, the membrane was then subjected to incubation in an ECL solution (Bio-Rad, Hercules, CA, USA). Increased chemiluminescence was used for observing protein signals. Quantitative values of the immunoblot assay were normalized to the sum of replicate methods [27].

We completed the normalization of the GFP signal using Quantity ONE to solve the loading control problem. First, the signal of the GFP band in Western blot (upper bands) and the signal of the loading proteins in the gel (lower bands) were quantified using Quantity ONE. Then, the ratio of the upper bands and the lower bands was profiled and analyzed for data significance.

## 3. Results

### 3.1. EWL Plant Expression Vector Construction

**Figure 1:**
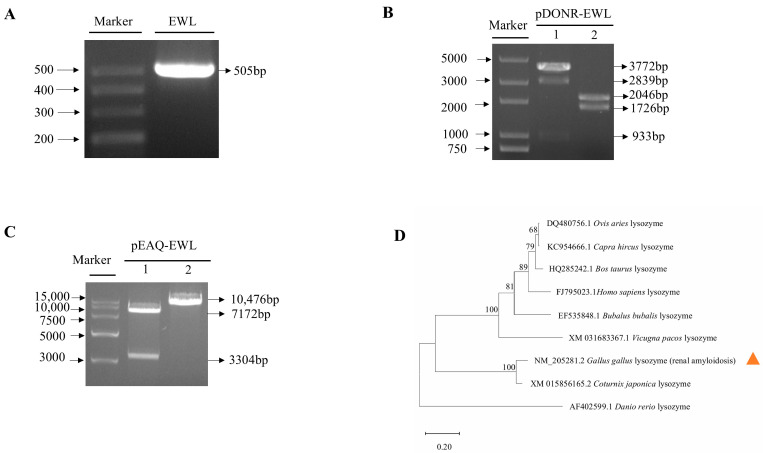
Synthesis and cloning of EWL. (**A**) Amplification of EWL gene by PCR. Marker, DL500 DNA Ladder. the 505 bp fragment is shown. (**B**) pDONR-EWL digestion. Marker, 2 kb plus DNA Ladder. Products following digestion by *Pst*Ⅰ and *Eag*Ⅰ are shown in lane 1 and 2, respectively. (**C**) pEAQ-EWL digestion. Marker, 15 k DNA Ladder. Products following digestion of pEAQ-EWL by *Spe*I and *ApaL*I are shown in lane 1 and 2, respectively. (**D**) EWL phylogenetic analysis. The neighbor-joining approach in the MEGA software (version 11.0) was used to build the phylogenetic tree based on the EWL sequences and lysozymes of various taxa. An orange marker designates the lysozyme in the hen egg white lysozyme.

Using pUC57-EWL plasmid as a template, a 505 bp band (444 bp of EWL plus attB1 and attB2) was amplified by PCR (Figure 1A). The sequence of the cloned EWL gene was identical to the sequence reported in the *Gallus* lysozyme (renal amyloidosis) (Figures S1 and S2). Specific bands of 1726 and 2046 bp were obtained by restriction endonuclease digestion using *Eag*Ⅰ/*Pst*Ⅰ (Figure 1B). The pEAQ-EWL plasmid was verified by restriction enzyme digestion with *Spe*I/*ApaL*I. The bands of 7172 and 3304 bp were obtained, suggesting that the pEAQ-EWL expression vector was correct (Figure 1C). The nucleotides sequence of the EWL gene was subjected to a BLAST search against public databases of the *Coturnix japonica* lysozyme, *Danio rerio* lysozyme, *Bos taurus* lysozyme, *Ovis aries* lysozyme, *Capra hircus* lysozyme, *Vicugna pacos* lysozyme, *Homo sapiens* lysozyme, and *Bubalus bubalis* lysozyme (Figure 1D). The hen egg white lysozyme (NM_205281.2) sequence was 100% identical to the *Coturnix japonica* lysozyme (XM_015856165.2); the *Vicugna pacos* lysozyme (XM_031683367.1) at the nucleotide level; only 68% identical to the *Ovis aries* lysozyme (DQ480756.1); and significantly different from the *Danio rerio* lysozyme (AF402599.1).

### 3.2. Conservation Analysis of EWL Lysozyme Among Different Species

**Figure 2:**
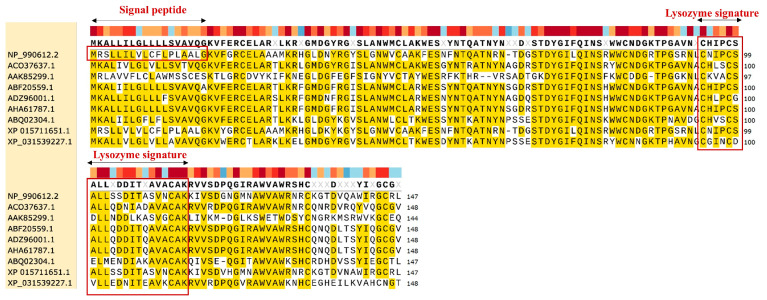
Alignment of protein sequence of EWL by ClustalW and SnapGene. Sequence conservation and consensus secondary structures are shown. Red squares represent the alpha-lactalbumin/lysozyme C signature and lysozyme activity position. Protein sequences from *Coturnix japonica* lysozyme (XP 015711651.1), *Danio rerio* lysozyme (AAK85299.1), *Bos taurus* lysozyme (ADZ96001.1), *Ovis aries* lysozyme (ABF20559.1), *Capra hircus* lysozyme (AHA61787.1), *Vicugna pacos* (XP_031539227.1) lysozyme, *Homo sapiens* (ACO37637.1) lysozyme, *Bubalus bubalis* (ABQ02304.1) lysozyme, *Gallus gallus* lysozyme (NP_990612.2) are from NCBI.

A quantity of 148 amino acids of EWL was obtained from GenBank: NM_205281.2. The physicochemical properties of the EWL protein were analyzed by the software of the ProtParam webserver (Table 1). The EWL protein has the chemical formula C_705_H_1116_N_214_O_204_S_12_ with a molecular weight of 16 kDa and 9.36 isoelectric points (Table S2). Based on the protein characterization database analyzed by InterPro, we identified that the EWL protein belongs to the Glycoside hydrolase family 22 and that it exhibits lysozyme activity [28]. An amino acid comparison of the enzymes among different species revealed that there were different degrees of mutations among different species. A 95% sequence similarity was found between the EWL (NP_990612.2) and the *Coturnix japonica* lysozyme (XP_015711651.1), and the similarity with other species was significantly reduced. These results suggest that, during the evolution of species, fewer mutations occurred between the same species and that more mutations occurred between different species (Figure 2).

The EWL protein sequence was analyzed by MotifScan [29]. We found that the motif of “MRSLLILVLCFLPLAALG” at positions 1–18 of the EWL protein sequence was the alpha-lactalbumin (lysozyme C) signature. The motif “CNIPCSALLSSDITASVNC” at positions 94–112 of the EWL protein sequence was identified as lysozyme activity.

### 3.3. Hen Egg White Lysozyme Is Structurally Related to Coturnix japonica Lysozyme and Bubalus bubalis Lysozyme

**Figure 3:**
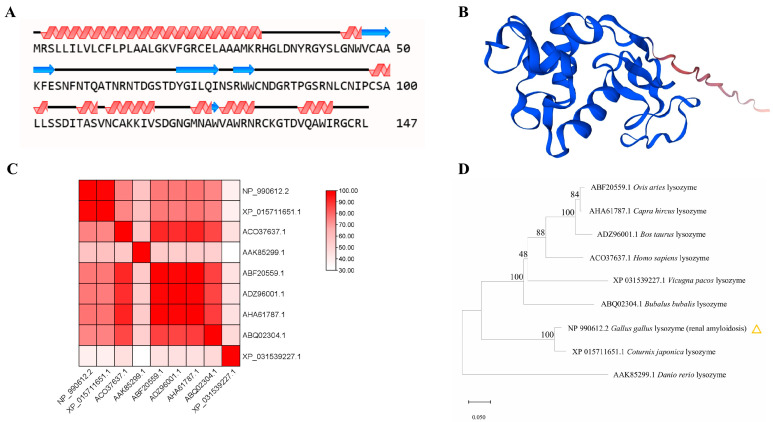
The prediction and analysis of EWL protein. (**A**) Secondary structures of EWL were predicted by NovoPro. The helix represents an alpha helix; the arrow represents a random coil; and the remainder represents the extended strand. (**B**) EWL tertiary structural models are predicted using Swiss-Model. (**C**) Structural similarity matrix between EWL and different proteins of other species. Prediction confidence scores are indicated in red color. (**D**) The phylogenetic analysis of EWL protein and other species. Values on branch are displayed. Protein sequences from *Coturnix japonica*, *Danio rerio*, *Bos taurus*, *Ovis aries*, *Capra hircus*, *Vicugna pacos, Homo sapiens*, *Bubalus bubalis* are from NCBI. An yellow marker designates the lysozyme in the hen egg white lysozyme.

The secondary structure of the EWL protein was analyzed by the software of NovoPro (Figure 3A). The alpha helix and random coil are the main structural elements of EWL. We predicted a three-dimensional structure of EWL using Swiss-Model and obtained a tertiary structure model with a GMQE value of 0.89 (Figure 3B).

To confirm that the cloned EWL protein is structurally related to known lysozymes, we performed comparative analyses of reference structures obtained by X-ray crystallography or high-resolution modeling. The structural similarity matrix and hierarchical clustering results indicated that the EWL (NP_990612.2) has spatial topologies close to those of the *Coturnix japonica* lysozyme (XP_015711651.1), which differed significantly from those of the *Vicugna pacos* lysozyme (XP_031539227.1) and the *Danio rerio* lysozyme (AAK85299.1) (Figure 3C,D).

### 3.4. Effect of Transient Expression of EWL in N. benthamiana on Plant Viral Infection

**Figure 4:**
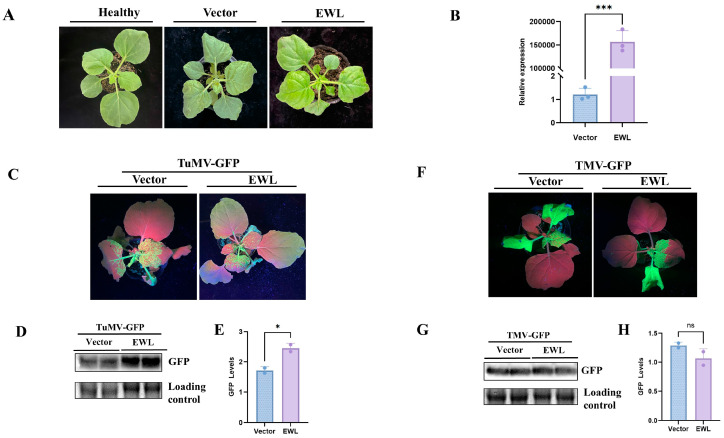
Expression of EWL protein and effect of transient expression of EWL on TuMV and TMV infection. (**A**) Plant phenotype in *N. benthamiana* after transient expression of EWL protein at 3 dpi. (**B**) EWL expression in agro-infiltrated leaves was detected by qPCR. (**C**) TuMV infection symptoms in *N. benthamiana* from transient expression of EWL. (**D**) Western blot analysis (anti-GFP) of TuMV viral accumulation. (**E**) Quantity ONE was used to quantify GFP signal. (**F**) TMV viral accumulation was detected by Western blot with anti-GFP. (**G**) Quantity ONE was used to quantify GFP signal. * represents difference at *p* < 0.05. (**H**) Quantity ONE was used to quantify GFP signal. *** represents significant difference at *p* < 0.001. ns represents no significance.

To verify the influence of the EWL protein on plant viral infections, we conducted transient expression assays in *N. benthamiana*. *Agrobacterium* strains C58C1 carrying pEAQ-EWL plasmids were infiltrated on *N. benthamiana* leaves. At 3 dpi, we observed no significant differences in plant phenotype compared with those of the control plants (heathy plants or empty vector) (Figure 4A). Meanwhile, the EWL protein expression in these samples was verified by RT-PCR, and the gene-specific bands were obtained (Figure S3). Correspondingly, high expression levels of the EWL protein were detected by qPCR (*p* < 0.01) (Figure 4B, Table S3).

Next, we performed the co-infiltration of pEAQ-EWL and TuMV-GFP into *N. benthamiana* to check the influence of EWL on viral infections. Symptoms of TuMV-GFP infection were observed at 7 d (Figure 4C). The GFP fluorescence in the upper part of the non-inoculated leaves was measured and photographed. Obviously, compared with the control, plants treated with EWL showed an increase in GFP fluorescence. Correspondingly, the TuMV-GFP viral accumulation was detected in plants co-infiltrated with EWL and TuMV-GFP by Western blot with anti-GFP. The results showed that viral accumulation significantly increased compared with the control (*p* < 0.05) (Figure 4D,E). Plants co-infiltrated with EWL and TMV-GFP did not show obvious differences in viral infection symptoms (Figure 4F). The viral accumulation of TMV-GFP detected by Western blot with anti-GFP showed no significant difference compared with those of the control plants (Figure 4G,H). The results reveal that overexpression of EWL specifically contributes to TuMV-GFP infection in *N. benthamiana* but not TMV-GFP infection (Table S4).

### 3.5. Effect of Transcient Expression of EWL in N. benthamiana on B. cinerea Infection

**Figure 5:**
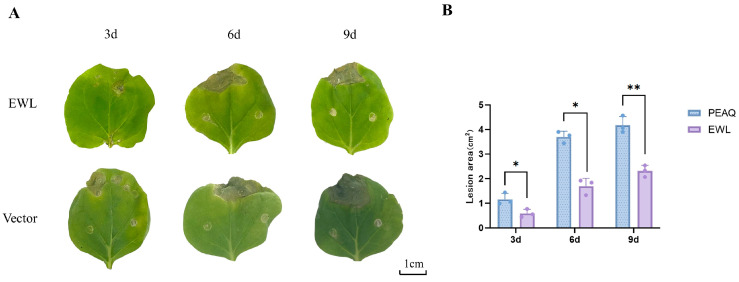
The effects of transient expression EWL on *B. cinerea* infection. (**A**) After *B. cinerea* inoculation, infection symptoms were observed at 3 dpi, 6 dpi, and 9 dpi. (**B**) The quantification of necrotic areas on *N. benthamiana* leaves infected with *B. cinerea*. * represents difference at *p* < 0.05. ** represents significant difference at *p* < 0.01. Scale bar is shown.

To examine if EWL could have an influence on fungal infection, *N. benthamiana* plants that transiently expressed EWL were inoculated with *B. cinerea*. Fungal infection symptoms were observed at 3 dpi, 6 dpi and 9 dpi (Figure 5A). The results showed that the necrotic spot areas of the EWL-treated leaves were smaller than those of the vector control. At 9 dpi, the necrotic spot areas of the leaves were significantly decreased (*p* < 0.01), suggesting that transcient expression of EWL was involved in the resistance of *N. benthamiana* against *B. cinerea* (Figure 5B, Table S5).

## 4. Discussion

Lysozyme has bactericidal immune function and is an important non-specific immune factor in organisms. When lysozyme is used in biology, it is more likely to be biologically modified to play a greater role; for example, the use of cloning technology to deeply analyze the biological structure of lysozyme can be accurately and efficiently used for the application of its characteristics. Lysozyme is used for fruit and vegetable preservation mainly through the formation of biofilm on its surface, which can effectively prevent microbial infections in the storage process, thus protecting the quality of fruits and vegetables during the storage period [30].

EWL is a natural antimicrobial protein with the advantages of strong bacterial inhibitory ability, safety and non-toxicity, and easy accessibility. In animal husbandry, EWL is added to animal feed instead of antibiotics in order to enhance the animal’s own immunity. EWL can significantly enhance the growth performance, bacteriostatic ability, and antioxidant properties of rabbits [31]. In recent years, EWL has been widely used in the fields of food, medicine, and animal husbandry, in addition to pesticide, biological, and chemical applications. EWL not only has multiple benefits for human health and livestock production but also improves plant resistance to pathogenic bacteria. The EWL gene was introduced into tobacco for the first time, and the transgenic plants were subsequently detected to have lysozyme activity [32]. The EWL gene was introduced into potato by transgenesis, and the results showed enhanced resistance to *Carotovorum* in the transgenic plants [33]. Transgenesis of the EWL gene into cotton revealed that EWL enhanced cotton resistance to Verticillium wilt by inhibiting fungal spread and producing ROS bursts [34]. EWL was also effective in inhibiting *Verticillium dahliae* and *Fusarium oxysporum* infestation with 75.7% fungal resistance [35]. Further evidence for the role of lysozyme content in reducing viral infections comes from studies on *Bombyx morii*, an insect that controls viral infections by significantly increasing overexpression of C-lysozyme, which contributes to antiviral immunity in the houseworm against infections from the nuclear polyhedrosis virus [36,37]. According to studies, lysozyme has a direct antiviral effect [6,19].

BmC-LZM was up-regulated in cells in response to viral infections [36]. In this study, we focus on the role of EWL on plant viral infections. We synthesized the EWL gene and constructed a plant expression vector. The presence of the lysozyme active site and signal site of EWL was confirmed by in silico analyses. EWL belongs to the Glycoside hydrolase family 22 and exhibits lysozyme activity. Alpha-lactalbumin and lysozyme type C belong to the Glycoside hydrolase family 22 and show enzymatic activity [38].

About 35% to 40% of the residues and the positions of the four disulfide bonds are the same in both proteins. Alpha-lactalbumin is a regulatory subunit of lactose synthase, and it can change N-acetylglucosamine to glucose. The lysozyme type C structure consists of a multi-domain mixed alpha and beta fold. These structures cleave peptidoglycan in the bacterial cell wall [38,39]. The EWL protein belongs to the C-type lysozyme/alpha-lactalbumin family and hydrolyzes chitin components of cells. This protein can hydrolyze the β-1,4-glycosidic bond formed between N-acetylglucosamine and N-acetylcytidylic acid in bacterial and fungal cell walls [40,41]. According to phylogenetic analysis, the EWL of *A. hypogaea* clustered with the *Coturnix japonica* lysozyme and the *Vicugna pacos* lysozyme, indicating that they may have similar activities.

We next investigated the role of EWL on plant viral infections. Interestingly, plants expressing EWL contributed to TuMV-GFP infection but not to TMV infection. The EWL gene may activate a relevant defense response in the plant after plant virus infestation of EWL-containing *N. benthamiana.* Or, perhaps this defense response is related to the structure and activity of EWL. Or, perhaps EWL directly interacts with the virus, thus inhibiting the amplification of the plant virus. It may be further hypothesized that lysozyme interacts with nucleic acids and alters their function, but whether this action is related to its catalytic activity or to its antimicrobial activity needs to be further investigated. Transient expression of the EWL gene can promote the accumulation of TuMV. This suggests that transient expression of EWL could affect plant antiviral defense, indirectly contributing to viral infection. Indeed, this issue has not yet been clearly or conclusively addressed, and further research should be carried out in the future.

We found that transient expression of EWL inhibited the fungal infection of *B. cinerea*. Therefore, the application of EWL in bioengineering could be promising in improving the disease resistance of plants. In recent years, the application of lysozyme has become more and more widespread; however, the production of lysozyme is an important factor limiting its application. Therefore, it is urgent to improve the production of lysozyme by using genetic engineering, chemical modification, and other methods. Although the amount of EWL in transient expression assays is relatively limited [42], it can still be used in plant bioreactors for the large-scale expression of EWL genes, which can save time and cost compared with the synthesis of lysozyme. Of course, when we want to obtain resistant plants, the EWL gene can be introduced into important crops by transgenic or gene-editing techniques. In this way, the problem of the difficulty in obtaining disease-resistant varieties through traditional breeding methods can be effectively improved.

## Figures and Tables

**Table 1:** Physicochemical parameters of EWL protein.

Parameter	EWL
Molecular formula	C_705_H_1116_N_214_O_204_S_12_
Molecular weight	16,238.65
Aliphatic coefficient	81.70
Instability index	19.86
Theoretical isoelectric point	9.36
Grand average of hydropathicity	−0.150
Total number of positively charged residues (Arg+Lys)	18
Total number of negatively charged residues (Asp+Glu)	9

## Data Availability

The original contributions presented in this study are included in the article. Further inquiries can be directed to the corresponding author(s).

## Supplementary Materials

The following supporting information can be downloaded at: https://www.mdpi.com/article/10.3390/life15040642/s1, Figure S1: Sequence map of EWL. Figure S2: Sequence of EWL. Figure S3: Specific band of EWL gene amplified by RT-PCR from infiltrated *N. benthamiana*. Table S1: Primers used in this study. Table S2: Amino acid composition of EWL proteins. Table S3: qPCR data for Figure 4B. Table S4: GFP signal of TMV-GFP and TuMV-GFP in Figure 4D,G quantified by Quantity ONE. Table S5: Area of necrotic spots on leaves infected by *B. cinerea*.
