# Supplementary material for: Transient Expression of Hen Egg White Lysozyme (EWL) in Nicotiana benthamiana Influences Plant Pathogen Infection
Source: Life (Basel). 2025 Apr 14;15(4):642. doi: 10.3390/life15040642 (PMC12028522; doi:10.3390/life15040642)
Supplement: Supplementary file 1 [file life-15-00642-s001.zip › life-3544639-supplementary.pdf]

Figure S1. Sequence map of LYZ.

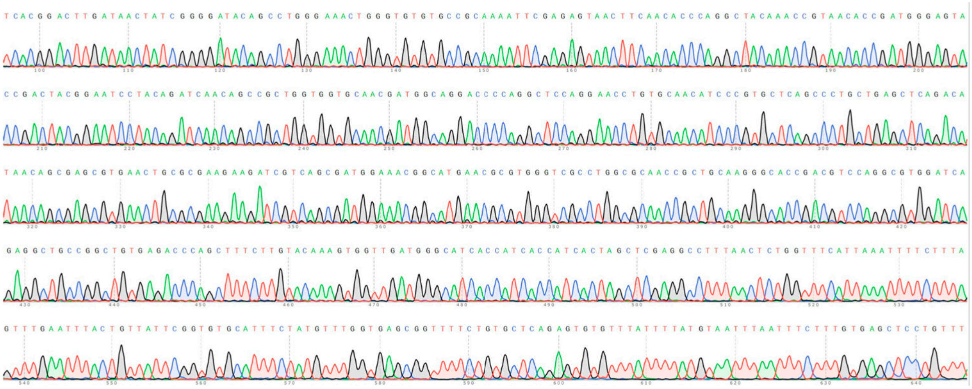

Figure S2. Sequence of LYZ.

5’ATGAGGTCTTTGCTAATCTTGGTGCTTTGCTTCCTGCCCCTGGCTGCTCTGGGGAAAG
TCTTTGGACGATGTGAGCTGGCAGCGGCTATGAAGCGTCACGGACTTGATAACTATCG
GGGATACAGCCTGGGAAACTGGGTGTGTGCCGCAAAATTCGAGAGTAACTTCAACAC
CCAGGCTACAAACCGTAACACCGATGGGAGTACCGACTACGGAATCCTACAGATCAAC
AGCCGCTGGTGGTGCAACGATGGCAGGACCCCAGGCTCCAGGAACCTGTGCAACATC
CCGTGCTCAGCCCTGCTGAGCTCAGACATAACAGCGAGCGTGAACTGCGCGAAGAAG
ATCGTCAGCGATGGAAACGGCATGAACGCGTGGGTCGCCTGGCGCAACCGCTGCAAG
GGCACCGACGTCCAGGCGTGGATCAGAGGCTGCCGGCTGTGA 3’

Figure S3. Specific band of LYZ gene amplified by RT-PCR from infiltrated *N. benthamiana*.

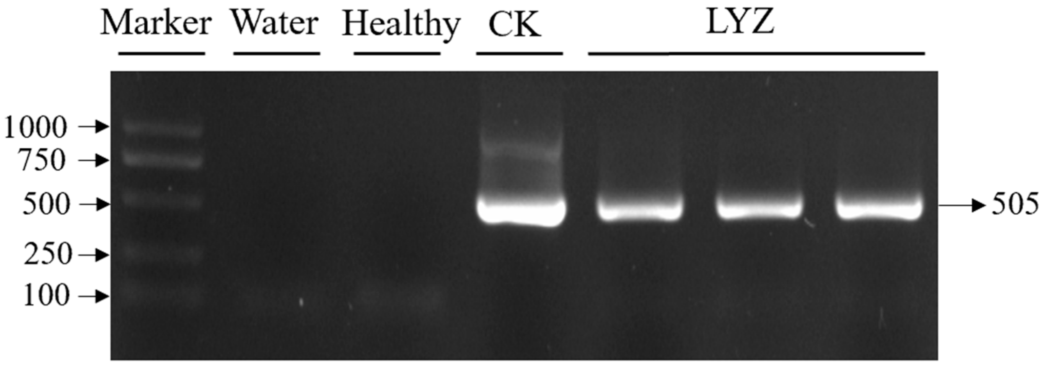

Table S1. Primers used in this study.

ID	Sequence(5′-3′)	Use
LYZ-F1	GGGGACAAGTTTGTACAAAAAAGCAGGCTTC ATGAGGTCTTTGCTAATCTTGGTGC	Cloning
LYZ-R1	GGGGACCACTTTGTACAAGAAAGCTGGGTCT CACAGCCGGCAGCCTC	
qPCR- LYZ -F2	TATGAAGCGTCACGGACTTG	RT-qPCR
qPCR- LYZ -R2	TTCACGCTCGCTGTTATGTC	
NtUB1-F	TCCAGGACAAGGAGGGTATCC	
NtUB1-R	GTCAGCCAAGGTCCTTCCATCC	

Table S2. Amino acid composition of LYZ proteins.

Amino Acid	RS	
	No. of Residues	Percentage of Residues
Ala (A)	14	9.5%
Arg (R)	12	8.2%
Asn (N)	14	9.5%
Asp (D)	7	4.8%
Cys (C)	9	6.1%
Gln (Q)	3	2.0%
Glu (E)	2	1.4%
Gly (G)	13	8.8%
His (H)	1	0.7%
Ile (I)	7	4.8%
Leu (L)	15	10.2%
Lys (K)	6	4.1%
Met (M)	3	2.0%
Phe (F)	4	2.7%
Pro (P)	3	2.0%
Ser (S)	11	7.5%
Thr (T)	7	4.8%
Trp (W)	6	4.1%
Tyr (Y)	3	2.0%
Val (V)	7	4.8%

Table S4. GFP signal of TMV-GFP and TuMV-GFP in Figure 4D and 4G quantified by Quantity ONE.

	TMV-GFP	TuMV-GFP
EWL	1.183056	2.340035
	0.949506	2.570194
Vector	1.250315	1.6296
	1.330287	1.801875

Table S5. Area of necrotic spots on leaves infected by *B. cinerea*

	3d	6d	9d
EWL	0.701	1.922	2.069
	0.552	1.84	2.33
	0.443	1.33	2.54
Vector	1.059	3.444	4.066
	0.99	3.9	3.9
	1.44	3.76	4.57
