# Supplementary material for: Transient Expression of Hen Egg White Lysozyme (EWL) in Nicotiana benthamiana Influences Plant Pathogen Infection
Source: Life (Basel). 2025 Apr 14;15(4):642. doi: 10.3390/life15040642 (PMC12028522; doi:10.3390/life15040642)
Supplement: Supplementary file 1 [file life-15-00642-s001.zip › Table S3.pdf]

Table S3. qPCR data for Figure 4B

		Target Ct value	Internal reference CT value	Target△Ct value	Average control△Ct	△△Ct	2-△△Ct		Average control△Ct	Average
Vector biological replicates (1)	technical replicates 1	30.70737839	17.81475449	12.8926239	12.67431323	0.2183107	0.8595714	1.0241317	12.67431323	12.65588
	technical replicates 2	30.64637756	17.75427628	12.89210129		0.2177881	0.8598828		12.34336599	
	technical replicates 3	30.2050457	17.96683121	12.23821449		-0.4360987	1.3529408		12.94995626	
Vector biological replicates (2)	technical replicates 1	30.28152084	17.86194801	12.41957283	12.34336599	0.0762068	0.9485483	1.1001282		
	technical replicates 2	30.97721291	17.90343857	13.07377434		0.7304084	0.6027333			
	technical replicates 3	29.5861187	18.0493679	11.53675079		-0.8066152	1.7491029			
Vector biological replicates (3)	technical replicates 1	28.99141502	17.83927727	11.15213776	12.45381482	-1.3016771	2.4651528	1.5176483		
	technical replicates 2	29.54037285	17.99035072	11.55002213		-0.9037927	1.8709781			
	technical replicates 4	32.52138519	17.8621006	14.65928459		2.2054698	0.2168141			
EWL biological replicates (1)	technical replicates 1	12.52935219	16.84058762	-4.311235428		-16.967114	128118.02	183743.14		
	technical replicates 2	12.39303017	16.82684708	-4.43381691		-17.089695	139479.68			
	technical replicates 3	12.0538063	17.51158714	-5.457780838		-18.113659	283631.71			
EWL biological replicates (2)	technical replicates 1	12.19853115	16.96510887	-4.766577721		-17.422456	175663.66	148128.27		
	technical replicates 2	12.17336178	16.78791046	-4.614548683		-17.270427	158094.45			
	technical replicates 3	12.81229877	16.91176033	-4.099461555		-16.75534	110626.7			
EWL biological replicates (3)	technical replicates 1	12.02283192	16.88160133	-4.858769417		-17.514648	187255.41	138238.7		
	technical replicates 2	12.38851547	16.74458313	-4.356067657		-17.011946	132161.84			
	technical replicates 4	12.68709564	16.57138824	-3.884292603		-16.540171	95298.844			
